# Enhanced sialylation of bone marrow mesenchymal stromal cells contributes to immune remodeling through macrophage polarization in multiple myeloma patients

**DOI:** 10.3389/fimmu.2026.1919303

**Published:** 2026-08-25

**Authors:** Achille Ruer, David Van Morckhoven, Karlien Pieters, Nathalie Meuleman, Laurence Lagneaux, Basile Stamatopoulos

**Affiliations:** 1 Translational Hematology and Oncology Research Laboratory, Jules Bordet Institute, Université Libre de Bruxelles (ULB), Brussels, Belgium; 2 Department of Hematology, Jules Bordet Institute, Brussels, Belgium

**Keywords:** macrophage, mesenchymal stromal cells, multiple myeloma, polarization, sialylation

## Abstract

**Introduction:**

In multiple myeloma (MM), the immune system is disrupted, partly due to tumor-modified bone marrow mesenchymal stromal cells (BM-MSCs). These MSCs show altered glycosylation, including increased sialylation, which may promote immune evasion through Siglec interactions.

**Methods:**

We quantified sialic acids and Siglec ligands on healthy donors-, MM-, T-MM-(treated multiple myeloma) and MGUS (monoclonal gammopathy of undetermined significance)-MSCs, and examined how different culture conditions (myeloma coculture, conditioned medium, hypoxia, inflammation, 3D culture) affect MSC glycosylation. Expression of 84 glycosylation-related genes was screened by qPCR. Macrophage polarization was then assessed after modulating MSC sialylation using inhibitors or an activator.

**Results:**

MM-MSCs exhibited significantly higher surface sialylation than HD-, T-MM-, and MGUS-MSCs. MM-MSC sialylation was positively correlated with the percentage of plasma cells and the LDH levels of MM patients. Direct MSC-plasma cell cocultures further enhanced hypersialylation compared with conditioned medium or transwell coculture, indicating a contact-dependent mechanism. qPCR screening identified two dysregulated genes, ST6Gal1 and NEU2, implicated in the sialylation pathway. Inflammation and hypoxia appeared as key players in the expression of Siglec-7L, Siglec-9L and ST6Gal1 by MSCs. Functionally, we showed that increased sialylation promoted an M2-like phenotype as measured by CD206, whereas inhibition shifted macrophages toward an M1-like profile as measured by CD40 and HLA-DR. We also showed that when MSC sialylation is inhibited, their ability to induce an M2 phenotype is reduced, as evidenced by the lower production of M2-associated cytokines.

**Conclusion:**

Our findings suggest that BM-MSC hypersialylation may contribute to immune remodeling in MM.

## Introduction

Multiple myeloma (MM) is the second most common hematological malignancy and is characterized by the clonal expansion of plasma cells (PCs) within the bone marrow (BM) (1, 2). It is often preceded by a premalignant, asymptomatic condition known as MGUS. Complex and bidirectional interactions between MM cells and the bone marrow microenvironment contribute to neoplastic plasma cell growth, proliferation, and drug resistance (3, 4). Among the various cellular components of the tumor microenvironment (TME), BM-MSCs and macrophages are key players (5, 6). Their crosstalk has been implicated in promoting immunosuppression, inflammation, and survival signals that support myeloma cell growth and dissemination (7, 8). However, the molecular mechanisms underlying this cell-to-cell communication remain poorly understood.

MSCs are multipotent cells originally described in the bone marrow (BM) (9), where they support hematopoiesis, maintain tissue homeostasis, and regulate immune responses. In MM, MSCs interact with malignant plasma cells, acquiring a tumor-supportive phenotype characterized by altered differentiation potential, a pro-inflammatory, senescent transcriptomic and secretory profile, as well as impaired immunomodulatory functions (10–13). MSCs also influence the behavior of innate immune cells, particularly macrophages, by modulating their activation and polarization states (14, 15). Their ability to induce M2-like immunosuppressive macrophages contributes to the establishment of an immune-permissive TME, which favors tumor growth and suppresses anti-tumor immune responses (16). Moreover, the hypoxic BM environment shapes MSC functions, promoting angiogenesis and treatment resistance that sustain their pro-tumoral activity (17, 18).

An important mediator of cell-to-cell interactions in the TME is glycosylation. More specifically, sialylation, the enzymatic addition of terminal sialic acids to glycoproteins and glycolipids by sialyltransferases, is a post-translational modification that regulates intercellular communication, cellular trafficking, signaling, and immune responses (19, 20). Altered sialylation patterns, particularly hypersialylation, have been reported in various malignancies, including MM, and are associated with immune evasion, tumor progression, and resistance to therapy (21, 22). In MM plasma cells, upregulation of α2,3- and α2,6-sialyltransferases, such as beta-galactoside alpha-2,3-sialyltransferase 6 (ST3GAL6) or beta-galactoside alpha-2,6-sialyltransferase 1 (ST6Gal1), has been linked to enhanced tumor cell adhesion, chemoresistance, and reduced susceptibility to natural killer (NK) cell-mediated cytotoxicity as well as a reduced overall survival (23–25). Notably, hypersialylated ligands can bind sialic acid-binding immunoglobulin-type lectins (Siglec) receptors expressed on the surface of immune cells, including macrophages, thereby inhibiting pro-inflammatory signaling and promoting an immunosuppressive phenotype (26, 27). While the role of sialylation in MM plasma cells has been previously explored (24, 25, 28), the sialylation profiles of MSCs derived from patients with MM and MGUS remain largely uncharacterized.

Macrophages exhibit remarkable plasticity within the TME, where they can adopt distinct functional states depending on local signals and cues (29). In MM, macrophages predominantly acquire an M2-like phenotype, characterized by the expression of immunosuppressive markers such as CD206, and the secretion of anti-inflammatory cytokines including IL-10 and TGF-β. This polarization contributes to immune evasion and promotes tumor progression within the bone marrow microenvironment (30, 31). MSCs have been shown to influence macrophage polarization through the release of soluble factors and extracellular vesicles (32, 33). Emerging evidence further highlights the critical role of glycan-mediated signaling in shaping macrophage fate (34, 35). Similar to checkpoint receptors, inhibitory Siglecs, upon binding to their respective ligands, recruit and activate the intracellular phosphatases SHP-1 and SHP-2 via their immunoreceptor tyrosine-based inhibitory motifs (ITIM) (36). Once activated, these phosphatases modulate signaling pathways (i.e. PI3K, MAPK, NF-κB) and reduce the production of pro-inflammatory cytokines (i.e. TNFα, IL-6) (37). In the context of MM, sialylated ligands expressed by stromal cells can engage inhibitory Siglec receptors on macrophages, thereby promoting their polarization toward an immunosuppressive, tumor-supportive M2 phenotype, ultimately leading to plasma cell expansion and disease progression (38).

Recent studies have highlighted that targeting the sialylation machinery (particularly sialyltransferases such as ST6Gal1 and ST3Gal6) can help to restore anti-tumor immunity across various cancers, including MM (23, 39). However, most investigations have focused on the sialylation patterns of malignant plasma cells, leaving the glycosylation landscape of the surrounding stromal compartment largely unexplored. Given that MSCs are a key component of the bone marrow niche, we hypothesized that their sialylation profile may play a pivotal role in modulating macrophage polarization and, consequently, in shaping the immune architecture of the TME.

In this study, we explored the functional impact of BM-MSC sialylation on macrophage polarization in the context of MM and MGUS. Using *in vitro* co-culture systems, glycosylation enzyme profiling, and macrophage functional assays, we dissect the influence of stromal cell sialylation on immune cell behavior. Importantly, we demonstrate that enhanced sialylation of BM-MSCs directly drives macrophage polarization toward an M2-like, tumor-promoting phenotype.

## Materials and methods

### Patient sample collection and preparation

This study was approved by the Jules Bordet Institute Ethics Committee and was conducted using bone marrow obtained with written informed consent from 103 MM patients, 21 MGUS patients and 60 healthy donors. MM patients were divided into two groups: untreated (samples collected at diagnosis, n=49) or treated (one or several lines of treatment, n=54). A summary of patients’ characteristics is presented in **Supplementary Data Table 1**.

### MSC culture conditions

Bone marrow was obtained by iliac crest aspirates and MSCs were isolated using the standard adhesion method. Additional details about culture conditions (13) and characterization (40) are detailed in **Supplementary Data Texts** and **Supplementary Figure S1**.

MSCs were grown in a 12-well plate for 24 hours (5 ×10^4^ cells per well) in 1 ml DMEM medium as described above. MSCs were cultured in different experimental conditions: co-culture with myeloma cell lines RPMI 8226 (ECACC, Salisbury, UK) or U266B1 (ATCC, Manassas, VA, USA) (10^5^ per well), with a 0.4 µm filter between MSCs and myeloma cells, and with conditioned medium (CM) from myeloma cells (500 µl DMEM + 500 µl CM). MSCs were seeded in the lower chamber and RPMI 8226 cells were placed in the upper chamber. Conditioned media were not filtered but were centrifuged twice prior to use: first at 800 × g to remove cells and then at 3,000 × g to remove cellular debris. The impact of an inflammatory environment on MSCs was evaluated as previously described (41). Briefly, cells were stimulated for 24h using a cocktail of pro-inflammatory cytokines: 25 ng/mL IL-1β (Miltenyi Biotec, Bergisch Gladbach, Germany), 50 ng/mL interferon gamma (IFN-γ) (RD Systems, Abingdon, United Kingdom), 50 ng/mL tumor necrosis factor alpha (TNF-α) (Miltenyi Biotec), and 10 ng/mL IFN-α (RD Systems). MSCs were also cultured in 3D spheroids using a Nunclon Sphera U-shaped-bottom microplate, allowing cells to grow without adhesion to the plastic.

Sialylation status of MSCs was artificially modified using two sialylation inhibitors, α2-3,6,8,9 Neuraminidase A (Bioké, Leiden, The Netherlands) and peracetylated 3-fluoroaxial N-acetylneuraminic acid (P-3F_AX_-Neu5Ac) (Bio-Techne, Minneapolis, MN, USA) and the sialylation activator *N*-Azidoacetylmannosamine-tetraacylated (Ac_4_ManNAz) (Sigma Aldrich). MSCs were pre-treated with the indicated compounds for 3 hours. Subsequently, the cells were washed 3 times with PBS to remove residual compounds before the addition of macrophages.

### Macrophage differentiation and polarization

Human monocytes were purified from peripheral blood mononuclear cells isolated from blood buffy coats (purchased at the Croix-Rouge de Belgique) after CD14 immunomagnetic positive selection (MicroBead Kit, Miltenyi Biotec) according to the manufacturer’s instructions. Purified monocytes were then differentiated into macrophages by the addition of Granulocyte-Macrophage Colony-Stimulating Factor (GM-CSF, RD Systems) at 25 ng/ml for 5 days in RPMI 1640 (Biowest, Nuailleí, France) supplemented with 10% of heat-inactivated fetal bovine serum (FBS, Sigma-Aldrich). Macrophages were polarized into M1 macrophages by incubation with 20 ng/ml of IFN-γ (R&D systems) and 100 ng/ml of LPS (#8630, Sigma-Aldrich), for 24h. Macrophage M2 polarization was obtained by incubation with 20 ng/ml of interleukin-4 (Tebu-Bio, Le Perray-en-Yvelines, France) and 20 ng/ml of interleukin-13 (Tebu-Bio) for 72h.

BM-MSCs and buffy coat-derived macrophages were co-cultured in direct contact for 24 h. For macrophage experiments, macrophages were generated from PBMCs obtained from 10 healthy donors, whereas MSCs were isolated from 19 healthy donors, 7 MM patients, 14 T-MM patients, and 3 MGUS patients. Co-culture experiments were performed using different combinations of MSC and macrophage donors according to cell availability. Co-culture supernatants were then collected to quantify cytokine levels and macrophages were harvested for flow cytometry assessment of specific cell surface antigens.

### Flow cytometry analysis (MSCs and macrophages)

Sialic acids on the surface of MSCs were detected using FITC-conjugated plant lectins *Sambucus nigra agglutinin* (SNA) and *Maackia Amurensis* Lectin II (MAL-II) (both from bioWORLD, Dublin, OH, USA). Siglec ligands (Siglec-7L and Siglec-9L) were labeled on MSCs using recombinant Siglec-7 and Siglec-9-Fc chimeras combined with an anti-Fc-PE antibody (Jackson ImmunoResearch Labs, West Grove, PA, USA). For SNA and MAL-II, data were expressed as the Mean Fluorescence Intensity Ratio (MFIR), calculated as the geometric mean fluorescence intensity of SNA- or MAL-II-stained cells divided by that of unstained cells. Macrophages were stained with anti-CD40-PE (clone REA73315C3, #130-110-946, Miltenyi Biotec) and anti-HLA-DR-PE-Cy5 (clone G46-6, #555813, BD Biosciences, Franklin Lakes, NJ, USA) antibodies for M1 polarization, and with anti-CD206-PE (clone DCN228, #130-124-233, Miltenyi Biotec) for M2 polarization. Given the low expression of CD40 and HLA-DR in M0 macrophages, they were quantified as MFIRs, reflecting changes in expression levels per cell, whereas CD206 was expressed as the percentage of positive cells, reflecting the appearance of an M2-like subpopulation. For CD40, HLA-DR, or CD206, the MFIR was calculated as the geometric mean fluorescence intensity of the specific marker divided by the geometric mean fluorescence intensity obtained with the corresponding isotype control (for CD40: isotype recombinant human IgG1- PE, clone REA293, #130-113-438, Miltenyi; for HLA-DR, mouse IgG2a κ isotype control Cy5, clone G155-178, #555575, BD Biosciences; for CD206, isotype control Ab mouse IgG1 PE, clone IS5-21F5, #130-113-200, Miltenyi). All flow cytometry assays were performed using the MACSQuant^®^ Analyzer 10 Flow Cytometer (Miltenyi Biotec). Gating strategy is detailed in **Supplementary Data Texts** and **Supplementary Figure S2**.

### qPCR screening

An explorative qPCR screening was performed on 3 samples of HD-, MGUS- and MM-MSCs using the Human Glycosylation RT^2^ Profiler PCR Array (Qiagen, Hilden, Germany). This plate includes 84 key genes encoding glycan processing enzymes such as glycosyltransferases and glycosidases involved in the metabolism of major sugars: galactose, glucose, mannose, N-acetylgalactosamine, N-acetylglucosamine, fucose and sialic acid. The kit was used according to the manufacturer’s protocol. Briefly, gene expression was normalized using reference genes with the most stable expression across the samples based on results from the entire array. All information (gene list, housekeeping genes used, Ct values and fold change values) concerning the screening are provided in **Supplementary Data Tables 2**–**4**.

### RNA extraction and quantitative PCR assays

Expression levels of genes of interest (including ST6Gal1 and NEU2) in MSCs were evaluated by real-time quantitative PCR (qPCR). Total RNA was extracted from BM-MSCs in a single step using TriPure Isolation Reagent (Roche Applied Science, Penzberg, Upper Bavaria, Germany). Additional details about reverse transcription and real-time PCR are available in **Supplementary Data Text** and primer sequences are provided in **Supplementary Data Table 5**.

### Enzyme-linked immunosorbent assay

ELISA kits were used to quantify secreted protein levels of TNF-α (RayBiotech, Peachtree Corners, GA, USA) in culture supernatants of primary macrophages, either co-cultured with MSCs or cultured alone, under M1 activation or control conditions. Similarly, ELISA kits were used to measure IL-10 (RayBiotech), TGF-β1 (RayBiotech) and CCL22 (RayBiotech) in supernatants from primary macrophages with or without MSC co-culture, under M2 activation or control conditions.

### Statistical analyses

Data are expressed as mean ± standard error of the mean (SEM). Comparisons between groups were assessed using the unpaired Mann-Whitney *U* test. For paired experiments, the Wilcoxon signed-rank test (two-tailed) was used. For the qPCR screening, the Student’s t-test was used. Differences were considered statistically significant at p < 0.05. All statistical analyses and graphical representations were performed using GraphPad Prism v.8.3 (GraphPad Software, San Diego, CA, USA).

## Results

### MM-MSCs express more sialic acids than HD-, T-MM- and MGUS-MSCs

**Figure 1:**
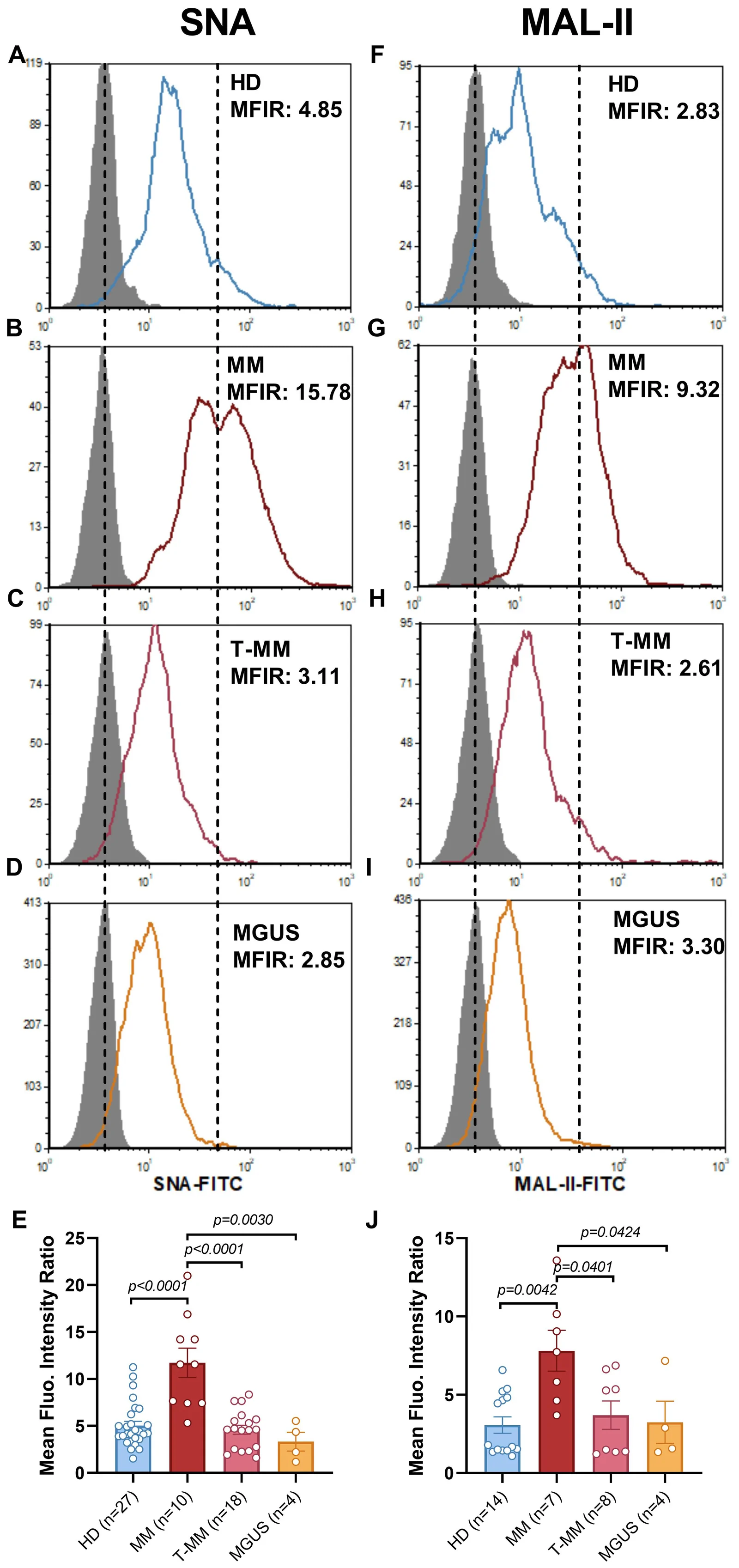
Sialylation is increased at the surface of MM-MSCs compared to HD-, T-MM- and MGUS-MSCs. BM-MSCs obtained from healthy donors **(A, F)**, patients diagnosed with MM **(B, G)**, treated patients with MM **(C, H)** and patients with MGUS **(D, I)** were stained with lectins SNA-FITC and MAL-II-FITC and measured by flow cytometry. SNA **(E)** and MAL-II levels **(J)** were compared between HD-, MM-, T-MM- and MGUS-MSCs. Graphs represent the mean fluorescence intensity ratio of BM-MSCs in flow cytometry as well as individual values. Pairwise comparisons were performed using the Mann–Whitney U test. n values are indicated in the figure or correspond to independent biological replicates. Data are presented as mean ± SEM.

Hypersialylation has been shown to play a role in immune evasion across many cancers, including colorectal, breast, lung, pancreatic and multiple myeloma (42, 43). However, BM-MSC sialylation remains poorly characterized within the bone marrow microenvironment, despite evidence that MSCs exhibit sialylation-related features (44, 45). Using flow cytometry, we first assessed the expression of sialic acids on the surface of MSCs with SNA and MAL-II lectins: we observed that sialylation detection by both SNA (**Figures 1A–E**) and MAL-II (**Figures 1F–J**) was significantly increased in MM-MSCs compared to HD-MSCs (SNA, p<0.0001; MAL-II, p=0.0042). Interestingly, SNA and MAL-II levels were statistically lower in T-MM-MSCs (p<0.0001 and p=0.0401, respectively) and MGUS-MSCs (p=0.0030 and p=0.0424, respectively) compared to MM-MSCs and not significantly different from HD-MSCs.

### Interaction with MM cells increases BM-MSC sialylation *in vitro* and is associated with clinical markers of MM

**Figure 2:**
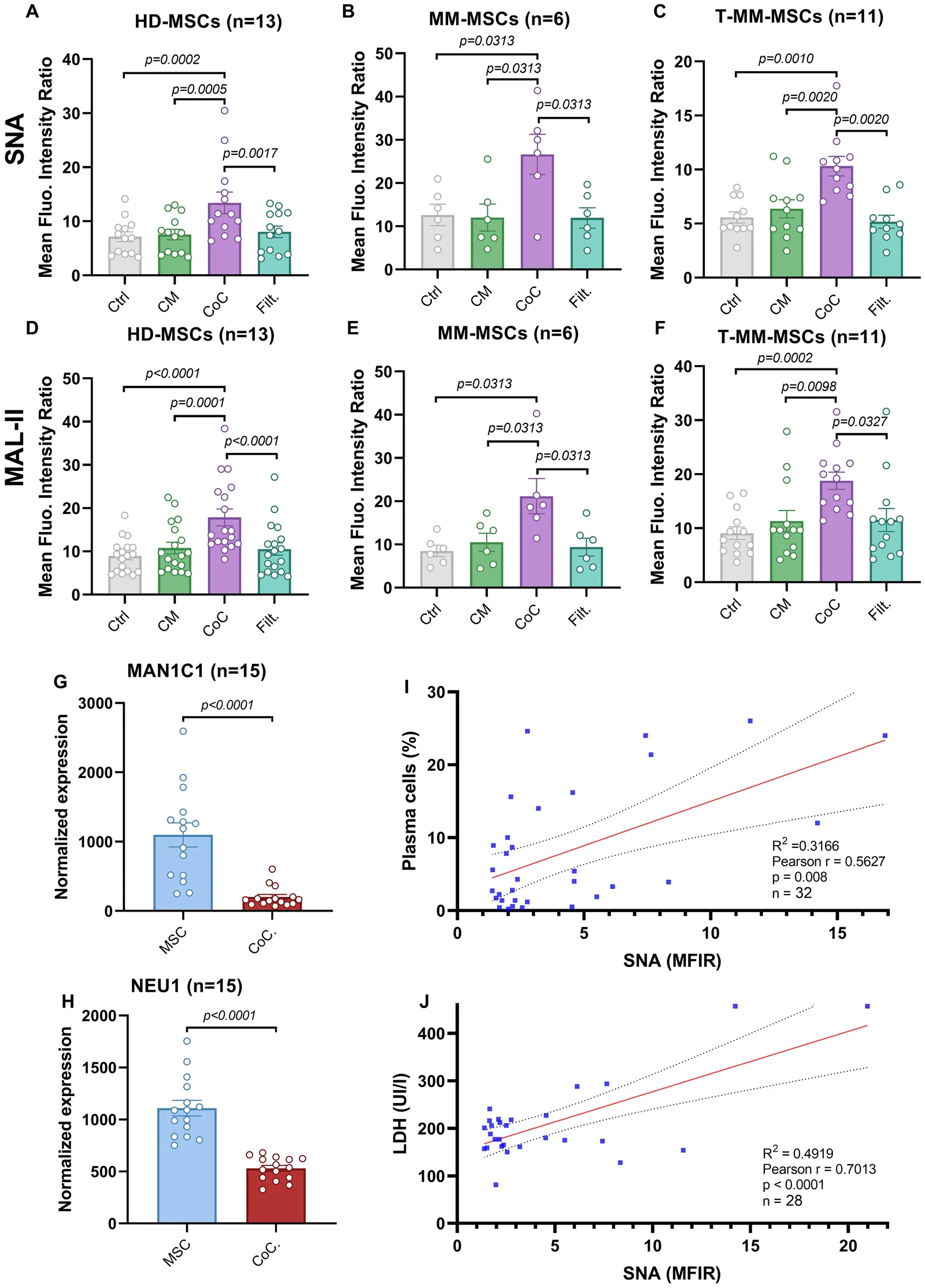
Interaction with MM cells increases BM-MSC sialylation *in vitro* and *in vivo.* For coculture experiments, BM-MSCs were obtained from healthy donors [**(A)**, n=13 and **(D)**, n=18], patients diagnosed with MM [**(B)**, n=6 and **(E)**, n=6] and treated patients with MM [**(C)**, n=11 and F, n=13] and were stained with lectins SNA-FITC **(A–C)** and MAL-II-FITC **(D–F)**. MSCs were cultured in 1 mL DMEM medium (Ctrl), in 500 µL DMEM + 500 µL conditioned medium of RPMI 8226 cell line (CM), in direct contact with RPMI cells (CoC) and in coculture with RPMI cells separated by a 0.4 µm filter (Filt.) for 24h. Data for MAN1C1 **(G)** and NEU1 **(H)** were obtained using publicly available data comparing the transcriptomic signature induced in MSCs alone or in coculture with malignant MM cells. Correlations between SNA binding on MM-MSCs and % of plasma cells in the bone marrow **(I)** and LDH levels **(J)**. Correlations were analyzed using Pearson’s test. Correlation coefficients (r), coefficients of determination (R^2^), p-values (p) and sample sizes (n) are indicated in each plot. Red lines represent the corresponding linear regression fits. Graphs represent mean fluorescence intensity ratio of BM-MSCs in flow cytometry **(A–F)**, normalized expression **(G, H)** and scatter plots **(I, J)** as well as individual values. Pairwise comparisons were performed using Wilcoxon matched-pairs signed-rank test. n values are indicated in the figure or correspond to independent biological replicates. Data are presented as mean ± SEM.

To assess the impact of myeloma cell contact and soluble factors on BM-MSC sialylation, HD, MM-, and T-MM-MSCs were cultured under four conditions: (1) DMEM alone, (2) conditioned medium from RPMI 8226 cells, (3) direct coculture with RPMI 8226 cells, and (4) trans-well coculture (0.4 µm). Surface sialylation on MSCs was analyzed by flow cytometry using SNA and MAL-II lectins and a CD166 labeling to distinguish MSC from myeloma cells. Our results showed a significant increase of sialylation detected by SNA binding on HD- (**Figure 2A**, p=0.0002), MM- (**Figure 2B**, p=0.0313) and T-MM-MSCs (**Figure 2C**, p=0.0010) cultured in direct contact with myeloma cells compared to MSCs cultured alone. Interestingly, similar increases were observed on MSCs in direct coculture (CoC) compared to MSCs cultured with RPMI 8226 conditioned medium (CM) or in coculture separated by a filter (Filt.). These results were further confirmed with MAL-II detection (**Figures 2D–F**). We verified that increased MSC sialylation also occurs in coculture with other MM cell lines (INF, MOL-2, EJM and U266) as described in **Supplementary Figure S3A**.

Using publicly available data comparing the transcriptomic signature induced in MSCs after interaction with malignant MM cells (n=15 paired experiments) (46), we screened the expression of the 84 key genes encoding glycan processing enzymes (**Supplementary Data Table S2**) and selected genes that were statistically significant in coculture condition compared to MSCs cultured alone and with a fold change higher than 2. We observed that MAN1C1 (Mannosidase Alpha Class 1C Member 1) was decreased by 6.7-fold (p<0.0001) and NEU1 (Neuraminidase 1) was decreased by 2.2-fold (p<0.0001) in coculture conditions (**Figures 2G, H**). These results are in line with our previous observations of increased SNA and MAL-II labeling on MM-MSCs, confirming the importance of cell-cell interactions between MSCs and MM cells on MSC sialylation. Our data highlights the importance of direct contact between MSCs and myeloma cells to observe MSC hypersialylation, since soluble factors from myeloma cells did not affect SNA nor MAL-II binding in HD-, MM- and T-MM-MSCs.

To assess whether the increased MM-MSC sialylation is associated with disease burden in MM patients, we next analyzed correlations between MM-MSC surface sialylation levels, quantified by SNA binding, and major clinical parameters of the present patient cohort for which clinical data were available. MM-MSC sialylation positively correlates with the percentage of bone marrow plasma cells (**Figure 2I**, R^2^ = 0.3166, p=0.0008), indicating an association with tumor infiltration. Moreover, MSC sialylation levels were associated with LDH levels (**Figure 2J**, R^2^ = 0.4919, p<0.0001), included in the *Revised International Staging System* (R-ISS). Together, these data suggest that MSC hypersialylation is indicative of disease burden in MM patients and strengthen its functional relevance within the TME.

### Inflammation modulates HD-MSC sialylation and Siglec-7 ligand and Siglec-9 ligand expression by HD- and MM-MSCs

BM-TME of patients with MM is characterized by inflammatory and hypoxic conditions (47, 48). We investigated the impact of inflammation and hypoxia on HD-MSC sialylation under both control conditions and during direct contact with the RPMI 8226 myeloma cell line. Additionally, we evaluated the effect of inflammation on MSC sialylation in three-dimensional culture conditions compared to the conventional two-dimensional monolayer culture.

**Figure 3:**
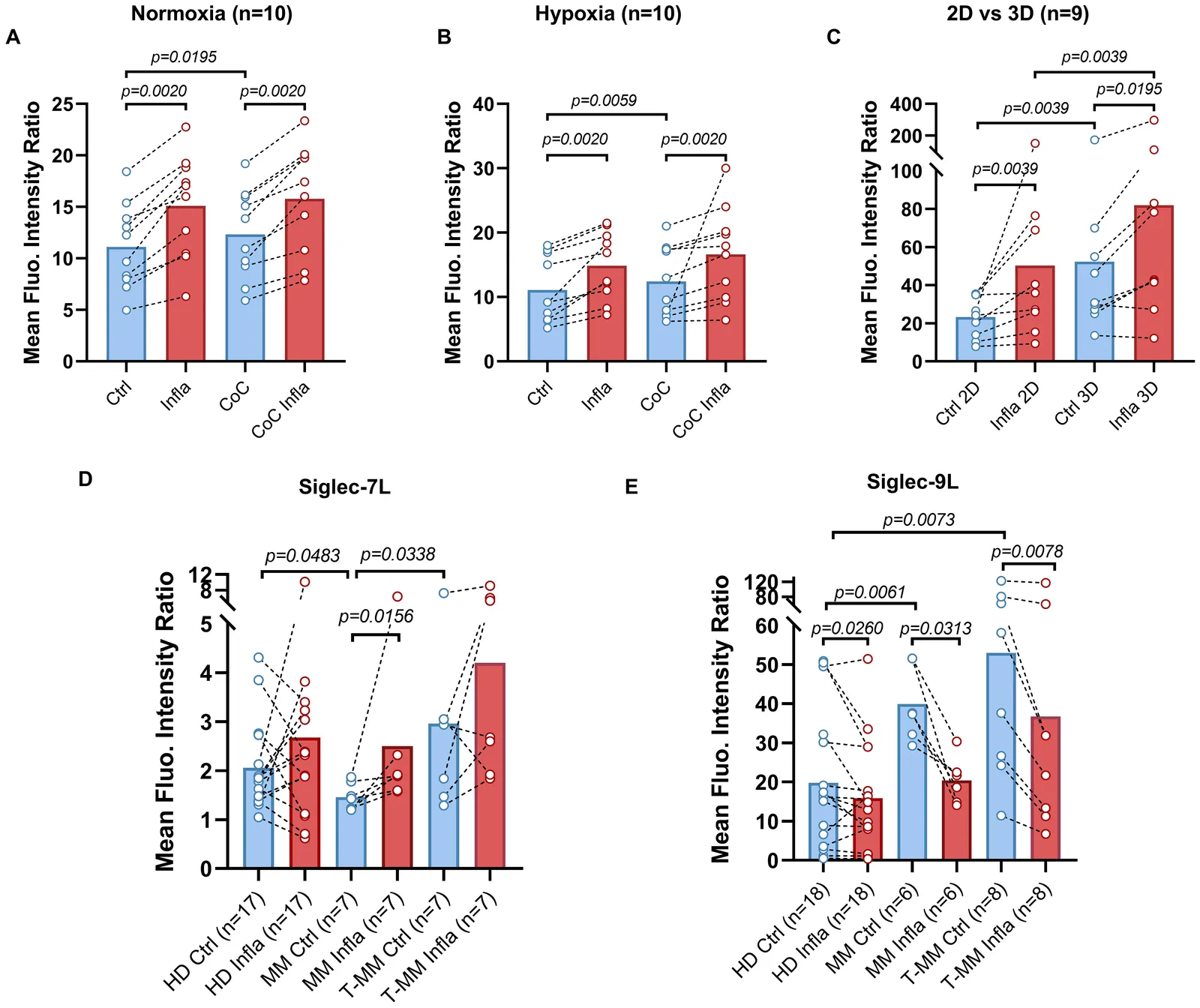
Inflammation increases HD-MSC sialylation in normoxic, hypoxic and 3D culture conditions and alters Siglec ligand expression. BM-MSCs obtained from healthy donors were cultured in 1mL DMEM (Ctrl) and 1mL pro-inflammatory medium (DMEM + IL-1β, IFN-γ, TNF-α, IFN-α, Infla). MSCs were cultured in normoxia [37 °C, 5% CO_2_, **(A)** n=10], hypoxia [37 °C, 1% O_2_, **(B)** n=10] and in a Nunclon Sphera U-shaped-bottom microplate, allowing cells to grow without adhesion to the plastic [**(C)** n=9]. MSCs were stained with lectin SNA-FITC. For Siglec ligand detection, BM-MSCs obtained from healthy donors, patients diagnosed with MM and treated patients with MM stained were with a recombinant Siglec-7 **(D)** and Siglec-9 **(E)** chimera protein linked with a PE antibody. MSCs were cultured in 1 mL DMEM (ctrl) and 1mL pro-inflammatory medium. Graphs represent the fluorescence intensity ratio of BM-MSCs in flow cytometry as well as individual values. Data were analyzed using the Wilcoxon signed-rank test for paired comparisons, including control versus inflammatory conditions, 2D versus 3D cultures, and control versus co-culture conditions. Comparisons between HD MSC, MM MSC, and T-MM MSC groups were performed using the Mann–Whitney U test. n values are indicated in the figure or correspond to independent biological replicates. Data are presented as mean ± SEM.

We showed that HD-MSCs cultured in inflammatory conditions generated by the addition of a pro-inflammatory cytokine cocktail (25 ng/mL IL-1β, 50 ng/mL IFN-γ, 50 ng/mL TNF-α, and 10 ng/mL IFN-α) display increased sialylation detected by SNA binding in control and coculture conditions both in normoxic (**Figure 3A**, p=0.0020 and p=0.0020) and hypoxic (**Figure 3B**, p=0.0020 and p=0.0020) environments. Interestingly, the difference in sialylation detected by SNA binding between MSCs cultured alone and those co-cultured with myeloma cells was no longer observed under inflammatory conditions, both in normoxia (p=0.1934) and hypoxia (p=0.6250). Moreover, HD-MSCs cultured in three-dimensional spheroids exhibit higher SNA binding compared to those grown in conventional two-dimensional monolayers, under both control (p=0.0039) and inflammatory conditions (p=0.0039) (**Figure 3C**). In spheroids, sialylation detected by SNA binding was also increased in HD-MSCs cultured with inflammation compared to the control condition (p=0.0195), consistent with previous findings in 2D cultures on the role of inflammation in MSC sialylation.

To further investigate BM-MSC sialylation, we measured the expression of ligands for Siglec-7 and Siglec-9 by HD-, MM- and T-MM-MSCs. The expression of Siglec-7L was higher both in HD-MSCs (p=0.0483) and in T-MM MSCs (p=0.0338) compared to MM-MSCs (**Figure 3D**). Inflammatory conditions induce a trend towards an increase of Siglec-7L in HD and T-MM and a significant increase in MM-MSCs (p=0.0156). For Siglec-9L, we observed a significantly higher expression in MM- (p=0.0061) and T-MM-MSCs (p=0.0073) compared to HD-MSCs (**Figure 3E**). Our results also indicate that Siglec-9L expression is decreased in HD- (p=0.0260), MM- (p=0.0313) and T-MM-MSCs (p=0.0078) cultured under inflammatory conditions.

### Sialylation enzyme-coding genes are differentially expressed in MM-MSCs compared to HD-MSCs

**Figure 4:**
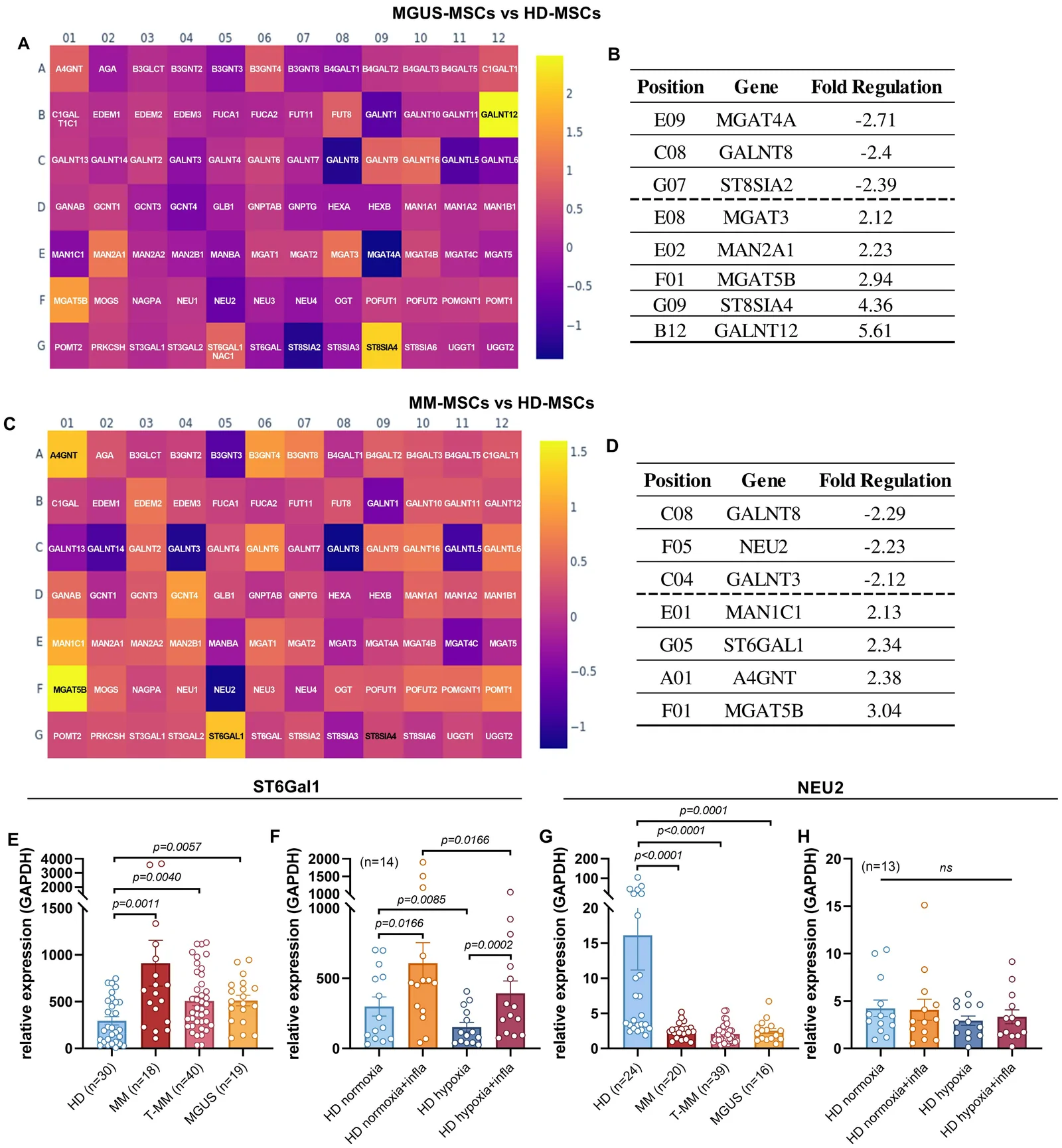
Impact of inflammation and hypoxia on differentially expressed genes in MM-MSCs compared to HD-MSCs. Eighty-four genes were screened and their expression was compared between MM- and HD-MSCs **(A)** and between MGUS- and HD-MSCs **(C)**. Differentially expressed genes were revealed in MM-MSCs **(B)** and MGUS-MSCs **(D)** (fold change > 2). RT-qPCR was performed on BM-MSCs obtained from healthy donors (blue), patients diagnosed with MM, treated patients with MM and patients with MGUS to detect ST6Gal1 **(E)** or NEU2 **(G)** transcript. RT-qPCR was also performed on HD-MSCs cultured in normoxia (37 °C, 5% CO_2_), hypoxia (37 °C, 1% O_2_) and pro-inflammatory medium (DMEM + IL-1β, IFN-γ, TNF-α, IFN-α, Infla) to detect ST6Gal1 **(F)** or NEU2 **(H)** transcript. ST6Gal1 and NEU2 transcripts were detected, using GAPDH as a housekeeping gene. Heatmaps represent the fold change for each transcript and graphs represent the mean of relative expression of ST6Gal1 and NEU2 normalized with GAPDH expression as well as individual values. Data were analyzed using the Student’s t-test for qPCR screening. Pairwise comparisons were performed for qPCR validation using Wilcoxon matched-pairs signed-rank test. n values are indicated in the figure or correspond to independent biological replicates. Data are presented as mean ± SEM.

In order to unravel the molecular aspects of BM-MSC sialylation, we performed an explorative qPCR screening of 84 glycosylation enzyme-coding genes, including sialylation enzymes such as sialyltransferases and neuraminidases on HD-, MM- and MGUS-MSCs (**Figures 4A, B**). Given the small number of samples screened for each type of MSC (n=3), we selected genes that had a fold change (FC) > 2 or < -2 to minimize false positive genes instead of using p-values (available in **Supplementary Data Table 6**). Two genes implicated in the sialylation process displayed a FC > 2 or < -2 in MM-MSCs compared to HD-MSCs: beta-galactoside alpha-2,6-sialyltransferase 1 (ST6Gal1, FC = 2.34), coding for an enzyme catalyzing the addition of sialic acid on proteins and neuraminidase 2 (NEU2, FC=-2.23), coding for a sialidase that hydrolyzes sialic acid bonds on glycoproteins. We confirmed by an individual qPCR on a higher number of samples that ST6Gal1 gene expression was significantly increased in MM- (p=0.0011), T-MM- (p=0.0040) and MGUS-MSCs (p=0.0057) compared to HD-MSCs (**Figure 4E**). Conversely, NEU2 expression was significantly decreased in MM- (p<0.0001), T-MM- (p<0.0001) and MGUS-MSCs (p=0.0001) compared to HD-MSCs (**Figure 4G**).

Then, we determined the impact of inflammation and hypoxia on ST6Gal1 and NEU2 gene expression levels in HD-MSCs. MSCs were cultured in control and inflammatory conditions in normoxia and hypoxia. Our results revealed that HD-MSCs cultured in an inflammatory medium exhibit higher expression of ST6Gal1 both in normoxic and hypoxic conditions (**Figure 4F**, p=0.0166 and p=0.0002 respectively). We also demonstrated that ST6Gal1 expression was lower in HD-MSCs cultured in hypoxic conditions, with or without inflammation (**Figure 4F**, p=0.0085 and p=0.0166 respectively). No significant impact of inflammation and hypoxia was observed on NEU2 expression in HD-MSCs (**Figure 4H**).

### MM-MSCs are more efficient at modifying M1 and M2 macrophage polarization

As previously shown, BM-MSCs express Siglec-7L and Siglec-9L, ligands for receptors that are located at the surface of immune cells, such as macrophages (37). We aimed to establish a link between BM-MSC sialylation and macrophage polarization by performing cocultures of BM-MSCs with M0 or M1 macrophages. We validated specific markers for M1 and M2 macrophages either by flow cytometry (CD40, HLA-DR for M1; CD206 for M2) or ELISA (IL10, CCL22, TGF-β for M2; TNFα for M1) (**Supplementary Figure S4***).*

**Figure 5:**
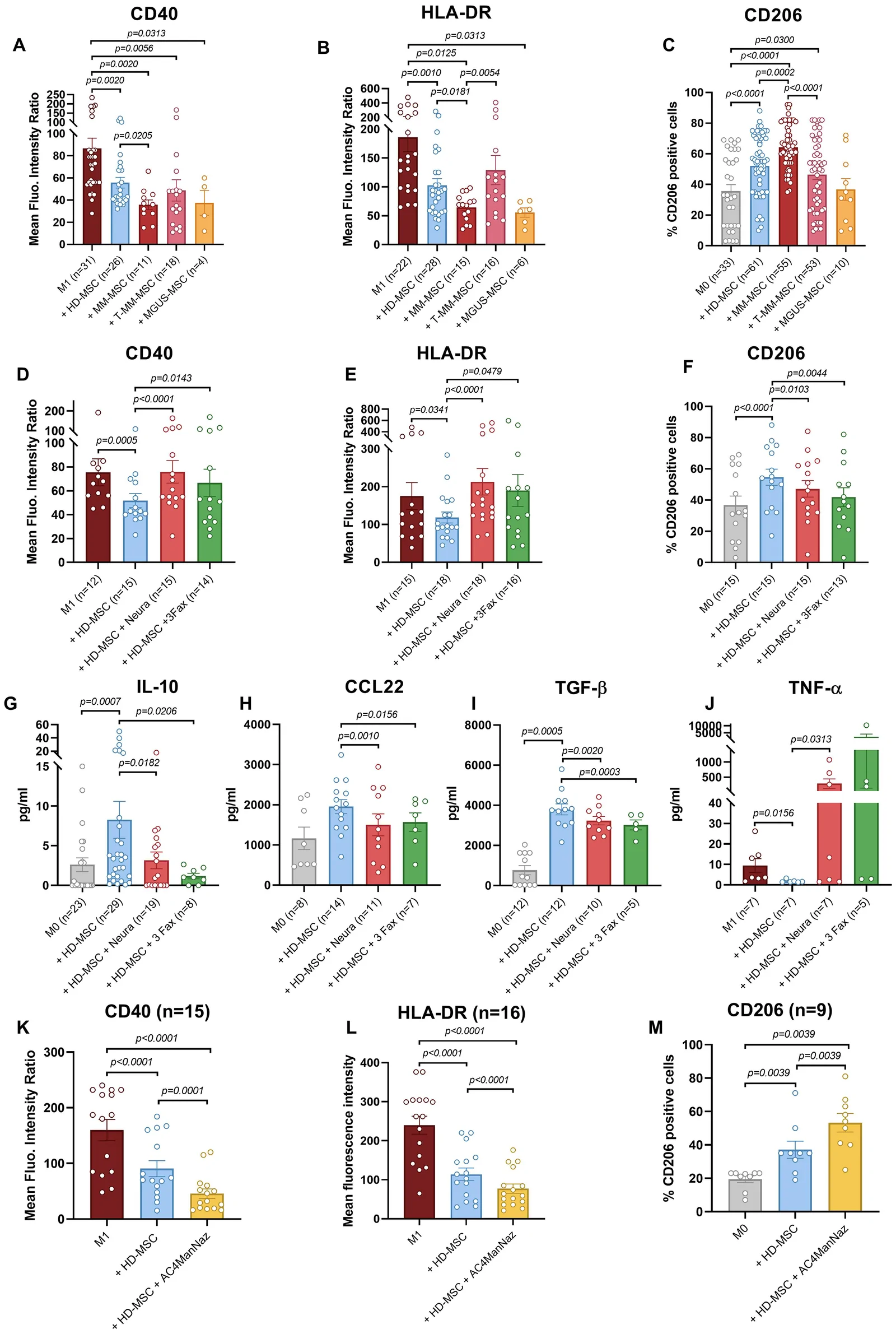
Inhibition or enhancement of BM-MSC sialylation differentially modulates macrophage polarization. M1 **(A, B)** and M0 **(C)** macrophages were cocultured with BM-MSCs obtained from healthy donors (blue), patients diagnosed with MM, treated patients with MM and patients with MGUS. Macrophage polarization was studied by flow cytometry detecting M1 markers CD40 **(A)** and HLA-DR **(B)** and M2 marker CD206 **(C)**. Sialylation of HD-MSCs was inhibited using two sialylation inhibitors: neuraminidase A and P-3F_AX_-Neu5Ac in coculture with M1 and M0 macrophages. Expression of M1 markers CD40 **(D)**, HLA-DR **(E)** and M2 marker CD206 **(F)** was assessed by flow cytometry using specific antibodies. M2 cytokines IL-10 **(G)**, CCL22 **(H)** and TGF-β **(I)** and M1 cytokine TNF-α **(J)** were also quantified in conditioned media from the cocultures by ELISA. Sialylation of HD-MSCs was enhanced by incubating them with Ac4ManNAz. Expression of CD40 **(K)**, HLA-DR **(L)** and CD206 **(M)** was assessed by flow cytometry using specific antibodies on M1 and M0 macrophages in coculture with HD-MSCs. Graphs represent the fluorescence intensity ratio for CD40 and HLA-DR and % of positive cells for CD206 as well as individual values. Graphs G, H, I and J represent mean concentration in pg/ml of cytokines IL-10, CCL22, TGF-β and TNF-α as well as individual values. For improved readability, only unique control values for macrophages cultured alone are displayed in the main figures, whereas all paired data points can be found in the supplementary figure (**Supplementary Figures S5**–**S8**). Pairwise comparisons were performed using Wilcoxon matched-pairs signed-rank test. n values are indicated in the figure or correspond to independent biological replicates. Data are presented as mean ± SEM.

First, HD-, MM-, T-MM and MGUS-MSCs were cocultured with M1-differentiated macrophages: we observed that CD40 and HLA-DR expression was decreased in M1 macrophages cocultured with HD- (p=0.0020 and p=0.0010 respectively), MM- (p=0.0020 and p=0.0125), T-MM- (CD40, p=0.0056) and MGUS-MSCs (HLA-DR, p=0.0313) compared to M1 macrophages cultured alone (**Figures 5A, B**). Interestingly, MM-MSCs induced a stronger decrease of the M1 phenotype compared to HD-MSCs (CD40, p=0.0205 and HLA-DR, p=0.0181) but this effect was lost when T-MM-MSCs were used (CD40, p=0.0918 and HLA-DR, p=0.5314). We also observed that coculture with MGUS-MSCs had already shown a capacity to modulate macrophage polarization since CD40 and HLA-DR levels are significantly lower compared to M1 macrophages cultured alone (CD40, p=0.0313 and HLA-DR, p=0.0313) but not statistically different from MM-MSC coculture (CD40, p=0.4063 and HLA-DR, p=0.1563).

To study the M2 phenotype, we utilized M0 macrophages as it has been previously described that MSCs in coculture with macrophages already transform them into M2 macrophages (49). CD206 expression was higher in M0 macrophages in coculture with HD- (p<0.0001), MM- (p<0.0001) and T-MM-MSCs (p=0.0300) compared to M0 macrophages cultured alone (**Figure 5C**). This increase was even higher when MM-MSCs were used relative to HD-MSCs (**Figure 5C**, p=0.0002). When T-MM-MSCs were used, CD206 expression was significantly decreased compared to coculture with MM-MSC (p<0.0001) suggesting that T-MM-MSCs are less able to induce an M2 phenotype.

### Modifying sialylation at BM-MSC surface directly impacts macrophage polarization

To determine the role played by BM-MSC sialylation in the modulation of macrophage polarization, we artificially modified HD-MSC sialylation levels in coculture with M1 and M0 macrophages using two sialylation inhibitors, α2-3,6,8,9 Neuraminidase A (Neura) and P-3F_AX_-Neu5Ac (3Fax) and one sialylation enhancer, *N*-Azidoacetylmannosamine-tetraacylated (Ac_4_ManNAz). As also shown above, the coculture of M1 macrophages with HD-MSCs induces a decrease of M1 marker levels (CD40, p=0.0005 and HLA-DR, p=0.0341). These decreases were abolished in M1 macrophages when HD-MSC sialylation was inhibited by incubation with Neura and 3Fax (**Figures 5D, E**): indeed, CD40 and HLA-DR were statistically increased after treatment with Neura (CD40, p<0.0001 and HLA-DR, p<0.0001) or 3Fax (CD40, p=0.0143 and HLA-DR, p=0.0479) compared to M1 macrophages cultured with HD-MSCs without sialylation inhibitors. On the contrary, we confirmed that CD206 expression is increased in M0 macrophages cultured with HD-MSCs (p<0.0001) compared to M0 macrophages alone (**Figure 5F**). Treating HD-MSCs with Neura or 3Fax showed a significant decrease of CD206 (**Figure 5F**, p=0.0103 and p=0.0044) compared to M0 macrophages cultured with HD-MSCs without sialylation inhibitors.

To validate that BM-MSC sialylation modulates macrophage polarization towards an M2 phenotype, we quantified M2-specific cytokines IL-10, CCL22 and TGF-β in culture supernatants from cocultures of M0 macrophages with HD-MSCs treated with sialylation inhibitors Neura and 3Fax. We showed that levels of IL-10, CCL22 and TGF-β were significantly lower in cocultures when HD-MSC sialylation was inhibited with Neura (IL-10, p=0.0182 and CCL22, p=0.0010) and 3Fax (IL-10, p=0.0206, CCL22, p=0.0156 and TGF- β, p=0.0020) compared to cocultures without sialylation inhibitors (**Figures 5G–I**). Interestingly, both Neura and 3Fax treatment restored a level of IL-10 (p=0.1934 and p=0.2188) and of CCL22 (p=0.3125 and p=0.6250) comparable to M0 macrophages cultured alone. For TGF-β, its level was reduced by Neura (p=0.0020), while after 3Fax treatment it remained higher (p=0.0003) compared to M0 macrophages cultured alone. Moreover, we observed decreased levels of TNF-α, an M1 cytokine, in supernatants of M1 macrophages in coculture with HD-MSCs compared to M1 macrophages cultured alone (**Figure 5J**, p=0.0313), confirming the anti-inflammatory effect of MSCs. Inhibiting MSC sialylation with Neura and 3Fax showed an increase or a trend of increase in TNF-α levels in M1 macrophages (**Figure 5J**, p=0.0313 for Neura, p=0.1250 for 3Fax), confirming that MSC sialylation suppresses the M1 phenotype of macrophages.

To further investigate the role of BM-MSC sialylation in macrophage polarization, we enhanced sialic acid biosynthesis at the surface of HD-MSCs using Ac_4_ManNAz. We verified that Ac_4_ManNAz effectively increases MSC sialylation by incubating them with different concentrations of Ac_4_ManNAz (**Supplementary Figure S3***)*. M1 macrophages exhibit lower expression of CD40 (p=0.0001) and HLA-DR (p<0.0001) when cocultured with HD-MSCs incubated with Ac_4_ManNAz (**Figures 5K, L**). We also showed that CD206 expression was higher on M0 macrophages in coculture with HD-MSCs incubated with Ac_4_ManNAz (p=0.0039) compared to M0 macrophages in coculture with HD-MSCs without Ac_4_ManNAz (**Figure 5M**). The paired comparisons corresponding to all panels of **Figure 5** are presented in **Supplementary Figures S5**–**S8**.

## Discussion

In MM, the immune system is impaired at multiple levels, and the bone marrow TME plays a pivotal role in both disease progression and therapy resistance. We and others have previously shown that MSCs undergo profound functional alterations within the MM bone marrow niche, thereby promoting tumor growth and contributing to immune dysregulation (10–13, 50). Here, we identify enhanced BM-MSC sialylation as a previously unrecognized mechanism driving macrophage polarization in the MM microenvironment.

Previous studies examining sialylation have focused primarily on malignant plasma cells. Glavey et al. reported that β-galactoside α-2,3-sialyltransferase (ST3GAL6) is highly expressed in MM cell lines and patients, altering transendothelial migration *in vitro* as well as homing and survival *in vivo* (25). Given the importance of MM cell–MSC interactions within the bone marrow TME, we investigated whether dysregulated sialylation also affects immunomodulatory functions of BM-MSCs. First, we observed that MM-MSCs display significantly elevated levels of α2,3- (MAL-II lectin) and α2,6-linked (SNA lectin) sialic acids compared with HD-MSCs. Consistent with our findings in MM, enhanced sialylation of tumor-conditioned stromal cells has also been reported in other malignancies, including colorectal and pancreatic cancers, where MSC-derived cancer-associated fibroblasts exhibit increased α2,3- and α2,6-linked sialic acids compared with normal stromal cells (38, 45, 51). Moreover, T-MM-MSCs and MGUS-MSCs exhibited reduced α2,6-linked sialic acids relative to MM-MSCs. This pattern likely reflects the absence of sustained stimulation by malignant plasma cells in treated MM or MGUS patients.

Supporting this interpretation, our coculture experiments demonstrated that direct contact with MM cells significantly enhances MSC sialylation. Natoni et al. demonstrated that inhibition of sialyltransferases diminishes MM cell binding to E-selectin, VCAM-1, and MADCAM-1, thereby disrupting integrin-mediated adhesion (23). Furthermore, analysis of publicly available transcriptomic data showed the decrease of two important glycan processing enzymes (MAN1C1 and NEU1) in MSCs when they are cocultured with MM cells. The downregulation of MAN1C1 after coculture with myeloma cells could impair the early trimming of mannose residues and potentially lead to altered glycan maturation. This imbalance could favor the formation of glycan structures that are more extensively sialylated, as sialyltransferases act on terminal positions of complex glycans. In addition, the decrease of NEU1, a neuraminidase removing sialic acids, will also contribute to an increased sialylation.

To transpose our findings *in vitro* into clinical relevance, we analyzed the relationship between MSC sialylation and disease-associated parameters in MM patients. MSC surface sialylation correlated positively with bone marrow plasma cell infiltration, and LDH levels, both markers of disease burden. Similarly, O’Neill et al. revealed that overexpression of the sialyltransferase ST6GALNAC6 and Siglec-10 ligands in the tumor stroma in CRC (colorectal cancer) was associated with lower overall survival of CRC patients (43). In MM, studies have shown associations between serum sialylation and the *International Staging System* (ISS), but not MSC sialylation (52, 53).

Inflammation is a key driver of disease progression and immune remodeling in MM (54, 55). We observed that α2,6-linked sialylation increases on HD-MSCs both when cultured alone and when cocultured with malignant plasma cells under inflammatory conditions. Interestingly, the increase in α2,6-sialylation detected by SNA binding in MSCs upon coculture with myeloma cells was abolished when MSCs were exposed to inflammatory conditions, suggesting that inflammation overrides the sialylation changes induced by direct tumor cell contact. A functional link between inflammation and sialylation has been demonstrated by Holdbrooks et al., who showed that ST6Gal1 modulates inflammatory signaling by prolonging NF-κB activation in response to TNF and LPS (56). Our findings are also consistent with O’Neill et al., who recently reported that inflammation reshapes the immune microenvironment in colorectal cancer and induces Siglec-G expression on macrophages and NK cells *in vivo* (43).

We further observed that inflammation tends to upregulate Siglec-7L levels in BM-MSCs, particularly in MM-MSCs, whereas Siglec-9L expression was significantly decreased in HD-, MM- and T-MM-MSCs under inflammatory conditions. However, it should be noted that SNA and MAL-II staining reflect global α2,6- and α2,3-sialylation, whereas Siglec-7L and Siglec-9L detect specific receptor-binding glycoepitopes. Consequently, increased overall sialylation does not necessarily result in increased expression of all Siglec ligands. The discordant regulation of Siglec-9L likely reflects qualitative changes in glycan composition and presentation rather than a reduction in cellular sialylation per se. Although modulation of MSC sialylation significantly altered macrophage polarization and changes in Siglec-7 and Siglec-9 ligand expression were observed under inflammatory conditions, the present study does not formally demonstrate that these effects are mediated by Siglec-dependent signaling. The importance of Siglec-7 and Siglec-9 receptors in reshaping the immune microenvironment has been shown in several malignancies such as pancreatic cancer, hepatocellular carcinoma and prostate cancer (34, 57, 58). More importantly, Daly et al. demonstrated high expression of Siglec-7L and Siglec-9L in MM cells, contributing to immune escape in the TME (24).

To further elucidate the molecular basis of MSC sialylation in MM, we performed an exploratory screening of 84 glycosylation enzyme-coding genes, including sialylation enzymes such as sialyltransferases and neuraminidases. Based on differential expression and biological relevance to the sialylation pathway, we selected and confirmed two differentially expressed enzyme-coding genes, ST6Gal1 and NEU2 in an independent set of samples. Functionally, ST6Gal1 catalyzes the addition of sialic acid in an α2,6-glycosidic linkage to the terminal galactose of N-glycoproteins, modulating a wide range of cell-cell and cell-matrix interactions. ST6Gal1 has been established as a key regulator in several cancers, including prostate, pancreatic, ovarian and colorectal cancers (59–62). In MM, its role remains largely unexplored, although Irons et al. reported its involvement in B cell-mediated suppression of medullary granulopoiesis (63). Here, we report the importance of ST6Gal1 expression in the regulation of MSC sialylation in the MM context, since we showed a significant increase in ST6Gal1 expression in MM-MSCs compared to HD-MSCs. Interestingly, although ST6GAL1 expression was increased in MGUS-MSCs, this was not associated with a significant increase in SNA binding, indicating that ST6GAL1 expression alone does not fully determine cell-surface α2,6-sialylation. As glycosylation is regulated by the coordinated activity of sialyltransferases, sialidases, substrate availability, and post-transcriptional regulatory mechanisms, additional alterations are likely required to generate the marked hypersialylation phenotype observed in MM-MSCs. On the contrary, NEU2 is a cytosolic sialidase involved in the regulation of intracellular sialylation. Although NEU2 is predominantly localized in the cytosol (64), several mechanisms may explain its influence on cell-surface sialylation. First, NEU2 can translocate to the plasma membrane under specific cellular conditions, allowing direct desialylation of membrane glycoproteins (65). Second, although this mechanism has not been directly demonstrated for NEU2, studies on NEU3 have shown that mammalian sialidases can localize to endosomal compartments and dynamically shuttle between endosomes and the plasma membrane (66). These observations suggest that sialidases may gain access to internalized glycoconjugates during intracellular trafficking, potentially contributing to the remodeling of cell-surface sialylation following receptor recycling. Third, NEU2 may indirectly influence cellular sialylation by regulating the turnover of intracellular sialylated glycoconjugates. Given its cytosolic localization and its role in the metabolism of sialylated substrates, alterations in NEU2 activity may affect the intracellular balance of sialylated molecules (67) and thereby contribute to broader changes in cellular glycosylation patterns. These mechanisms collectively provide a plausible explanation for the effects of NEU2 on cell-surface sialoglycans despite its predominantly cytosolic localization. Reduced NEU2 expression has been described in prostate and ovarian cancer cells (68, 69). In this context, we observed a decreased level of NEU2 in MM-, T-MM- and MGUS-MSCs relative to HD-MSCs.

Beyond inflammation, hypoxia is a critical factor in MM progression, immune evasion and therapy resistance (18, 48, 70). Previous studies have produced context-dependent effects of hypoxia on sialylation: Nawafley et al. observed increased expression of Siglec-7 ligands in a sub-population of K562 cells (71) under hypoxic stress, whereas Peng et al. reported reduced sialylation in breast cancer cells exposed to hypoxic conditions (72). Although the specific contribution of hypoxia to MSC sialylation remains poorly defined, our results suggest that ST6Gal1 expression is modulated by hypoxic stress in addition to inflammatory cues.

To determine how MSC sialylation contributes to this skewing, we examined its impact on macrophage polarization. Our data first showed that MSCs cocultured with macrophages inhibit M1 polarization and favor M2 polarization, with MM-MSCs exerting the most pronounced effect. The induction of macrophage polarization towards an M2 phenotype by MSCs has been previously described through cytokines and exosomes secretion and activation of immunomodulatory pathways (73–75). A limitation of this study is that macrophage polarization was assessed using a restricted panel of surface markers (CD40 and HLA-DR for M1 macrophages and CD206 for M2 macrophages). Although these markers are commonly used to characterize macrophage phenotypes, additional markers such as CD80/CD86 and CD163/CD209, together with high-dimensional phenotyping approaches, would provide a more comprehensive assessment of macrophage polarization. Nevertheless, the observed phenotypic changes were supported by cytokine analyses showing increased expression of TNF-α in M1 macrophages and IL-10, CCL22, and TGF-β in M2 macrophages, consistent with the expected functional polarization states.

Finally, pharmacologic targeting of sialylation using Neuraminidase A and P-3F_AX_-Neu5Ac reversed this phenotype and enhanced M1 polarization, whereas increasing sialic acid biosynthesis with Ac4ManNAz further promoted M2 polarization. Büll et al. demonstrated the impact of P-3F_AX_-Neu5Ac *in vivo* and showed that sialylation inhibition impairs adhesion, migration and *in vivo* tumor growth in a murine model of melanoma (76). Previous studies have shown that MSC sialylation promotes M2 polarization in cervical and colorectal cancer (43, 77) and O’Neill et al. demonstrated that targeting sialylation on stromal cells reverses macrophage suppression *in vivo* (43). Moreover, Barkal et al. described the role of the sialic acid/Siglec axis in inhibiting macrophage phagocytosis function via CD24 signaling (78). Finally, sialylation inhibition at the surface of MM cells has shown an enhancement of NK-cell mediated tumor cell lysis, confirming the role of sialylation in immune suppression in the TME of MM (24). In preclinical models, targeting sialylation with a selective sialyltransferase inhibitor significantly reduced cell migration and invasion *in vitro*. Moreover, in tumor-bearing mice, this approach decreased tumor size, angiogenesis, and metastatic potential, highlighting the promising therapeutic potential of such inhibitors for future clinical trials (79).

Although our findings, derived from primary human BM-MSCs, functional co-culture assays, and analyses of patient transcriptomic datasets, consistently support a role for MSC hypersialylation in shaping the immune microenvironment, future studies using syngeneic MM models or patient-derived xenografts will be required to establish its causal contribution to MM progression and to evaluate the therapeutic potential of targeting this pathway.

Our findings identify sialylation as a promising therapeutic target in MM. Pharmacological inhibition of sialyltransferases or enzymatic removal of sialic acids reversed M2 polarization and restored pro-inflammatory macrophage phenotypes *in vitro*, suggesting that disrupting the sialoglycan–Siglec axis could reprogram the bone marrow niche toward a more immunostimulatory state. Recent studies have demonstrated the feasibility of targeting this pathway in cancer, including the use of sialidase conjugates and small-molecule inhibitors to block Siglec–sialic acid interactions (80–82). In MM specifically, Ray et al. showed that interfering with Siglec-mediated checkpoints can reverse plasmacytoid dendritic cell–driven immunosuppression (83). Future studies should explore the integration of sialylation-targeted strategies with existing treatments to overcome resistance and enhance anti-myeloma immunity, particularly in combination therapies.

## Data Availability

The datasets presented in this study can be found in online repositories. The names of the repository/repositories and accession number(s) can be found in the article/**Supplementary Material**.

## In memoriam

Laurence Lagneaux passed away before the submission of this article. She made a significant contribution to this work. All co-authors attest to her involvement and have approved her inclusion as a co-author.
