# Supplementary material for: Enhanced sialylation of bone marrow mesenchymal stromal cells contributes to immune remodeling through macrophage polarization in multiple myeloma patients
Source: Front Immunol. 2026 Aug 25;17:1919303. doi: 10.3389/fimmu.2026.1919303 (PMC13550185; doi:10.3389/fimmu.2026.1919303)
Supplement: Supplementary file 1 [file SupplementaryFile1.docx]

**Supplemental Data**

**MSC culture conditions and Characterization**

Mononuclear cells were isolated by density gradient centrifugation (Pancoll, Palm Biotech, Aidenbach, Germany), washed in Hank’s buffered salt solution (HBSS, Lonza Europe, Verviers, Belgium) and seeded in Dulbecco’s modified Eagle’s medium–low glucose (DMEM-LG, Lonza) supplemented with 15% fetal bovine serum (FBS, Sigma-Aldrich, Saint-Louis, MO, USA), 2 mM L-glutamine and 1% penicillin-streptomycin (both from Lonza). Cells were incubated at 37°C in a 5% CO2 and 95% humidified atmosphere. After 48 h, non-adherent cells were removed by washing and the medium was changed twice a week. Thereafter, cells were detached with TrypLE™ Select solution (Lonza) and subcultured at 10^3^ cells/cm^2^ for their expansion by passage. MSCs were immunophenotypically characterized according to the ISCT criteria by assessing their adipogenic, osteogenic and chondrogenic lineage differentiation capacities, their adhesion to plastic and the presence/absence of specific surface markers by flow cytometry (Supplemental Figure S1)

**Reverse transcription and real-time PCR**

Reverse transcription was performed with 500 ng RNA using qScript Ultra SuperMix (Quanta Biosciences, Beverly, MA, USA). Quantification of transcripts was carried out by qPCR using 25 ng of cDNA, SYBR Green PCR Master Mix (Applied Biosystems, Foster City, CA, USA) and 0.32 µmol/L of each forward and reverse primer. GAPDH gene served as the housekeeping gene for normalization. The reactions were run on a ViiA 7 Real-Time PCR System (Applied Biosystems). Data were analyzed using the comparative ΔΔCt method and the fold changes were then calculated as fold change=2^–ΔΔCt^. Primer sequences are provided in Supplemental Data Table 5.

**Flow cytometry gating strategy**

Flow cytometry analyses were performed after exclusion of doublets using FSC-A versus FSC-H gating. Debris was subsequently excluded based on FSC and SSC characteristics to define the cell population of interest. The percentage of positive cells or the MFIR was quantified within this gated population. For SNA and MAL II staining, unstained cells were used as negative controls to define positive populations. For CD40, HLA-DR, and CD206 staining, the corresponding isotype controls were used to establish positivity thresholds. A representative gating strategy is provided in Supplemental data Figure S

**Supplemental Data Figure Legends**

**Figure S1: Characterization of BM-MSCSs.** (A) Cell surface marker expression on BM-MSCs was evaluated by flow cytometry. (B) Cell differentiation potential of MSCs was analyzed: (a) undifferentiated MSC observed by phase contrast microscopy (magnification x100). After 2-3 weeks in respective induction media, MSC were stained positively for lipid vacuoles with Oil Red O (x100) (b) showed the formation of mineralized matrix assessed by Alizarin red staining (x100) (c) and were positive for glycosaminoglycan matrix stained by Alcian blue (x25) (d), indicating adipogenic, osteogenic and chondrogenic differentiation respectively.

**Figure S2. Flow cytometry gating strategy.** The flow cytometry analysis was performed using the following gating strategy. (A) doublets were excluded based on forward scatter area (FSC-A) versus forward scatter height (FSC-H) parameters to retain only single cells. (B) debris was removed by gating the main cell population according to its forward scatter (FSC) and side scatter (SSC) characteristics. (C) the percentage of positive cells or the MFIR was then determined within the gated cell population.

**Figure S3: SNA binding on MSCs in coculture with MM cell lines and incubated with Neuraminidase A, P-3F_AX_-Neu5Ac and Ac4ManAz.** (A) HD-MSCs in coculture with different myeloma cell lines. (B) Neuraminidase A and P-3F_AX_-Neu5Ac were incubated with HD-MSCs at 200 u/ml and 200 µM respectively. (C) Ac4ManAz was incubated with HD-MSCs at 5 different concentrations: 0 µM, 50 µM, 100 µM, 200 µM and 400 µM. SNA lectin level was measured by flow cytometry. Data were analyzed using the Wilcoxon signed-rank test. Graphs represent the MFIR and % control of SNA binding as well as individual values.

**Figure S4: M1 and M2 markers for flow cytometry assays.** M1 markers CD40 (A) and HLA-DR (B) and M2 marker CD206 (C) measured by flow cytometry on M0, M1 and M2 macrophages. M2 markers IL-10 (D), CCL22 (E) and TGF-β (F) and M1 marker TNF-α (G) dosed by ELISA assays on M0 and M2 macrophages. Data analyzed using Wilcoxon signed-rank test. Graphs A, B and C represent mean fluorescence intensity for CD40 and HLA-DR and % of positive cells for CD206 as well as individual values. Graphs D, E and F represent the mean concentration in pg/ml of cytokines IL-10, CCL22 and TGF-β as well as individual values.

**Figure S5: Impact of MSC co-culture on macrophage polarization assessed by a CD40, HLA-DR and CD206 staining.** M1 and M0 macrophages were cocultured with BM-MSCs obtained from healthy donors (blue), patients diagnosed with MM, treated patients with MM and patients with MGUS. Macrophage polarization was studied by flow cytometry detecting M1 markers CD40 (A) and HLA-DR (B) and M2 marker CD206 (C). Paired comparisons are shown by horizontal lines. Data were analyzed using the Wilcoxon signed-rank test. Graphs represent the fluorescence intensity ratio for CD40 and HLA-DR and % of positive cells for CD206 as well as individual values.

**Figure S6: Inhibition of BM-MSC sialylation differentially modulates macrophage polarization towards an M1 phenotype.** M1 and M0 macrophages were cocultured with BM-MSCs obtained from healthy donors (blue), patients diagnosed with MM, treated patients with MM and patients with MGUS. Sialylation of HD-MSCs was inhibited using two sialylation inhibitors: neuraminidase A and P-3FAX-Neu5Ac in coculture with M1 and M0 macrophages. Expression of M1 markers CD40 (A), HLA-DR (B) and M2 marker CD206 (C) was assessed by flow cytometry using specific antibodies. Paired comparisons are shown by horizontal lines. Data were analyzed using the Wilcoxon signed-rank test. Graphs represent the fluorescence intensity ratio for CD40 and HLA-DR and % of positive cells for CD206 as well as individual values.

**Figure S7. Impact of MSC co-culture on macrophage polarization assessed by cytokine productions.** M1 and M0 macrophages were cocultured with BM-MSCs obtained from healthy donors (blue), patients diagnosed with MM, treated patients with MM and patients with MGUS. Sialylation of HD-MSCs was inhibited using two sialylation inhibitors: neuraminidase A and P-3FAX-Neu5Ac in coculture with M1 and M0 macrophages. M2 cytokines IL-10 (A), CCL22 (B) and TGF-β (C) and M1 cytokine TNF-α (D) were quantified by in conditioned media from the cocultures by ELISA assays. Paired comparisons are shown by horizontal lines. Data were analyzed using the Wilcoxon signed-rank test.

**Figure S8. Enhancement of BM-MSC sialylation differentially modulates macrophage polarization towards an M2 phenotype.** M1 and M0 macrophages were cocultured with BM-MSCs obtained from healthy donors (blue), patients diagnosed with MM, treated patients with MM and patients with MGUS. Sialylation of HD-MSCs was enhanced by incubating them with Ac4ManNAz. Expression of CD40 (A), HLA-DR (B) and CD206 (C) were assessed by flow cytometry using specific antibodies on M1 and M0 macrophages in coculture with HD-MSCs. Graphs represent the fluorescence intensity ratio for CD40 and HLA-DR and % of positive cells for CD206 as well as individual values. Paired comparisons are shown by horizontal lines. Data were analyzed using the Wilcoxon signed-rank test.

**Supplemental Figures**

**Supplemental Data Figure S1. Characterization of BM-MSCSs.**

**Supplemental Data Figure S2. Flow cytometry gating strategy.**

**Supplemental Data Figure S3. SNA binding on MSCs in coculture with MM cell lines and incubated with Neuraminidase A, P-3FAX-Neu5Ac and Ac4ManAz.**

**Supplemental Data Figure S4. M1 and M2 markers for flow cytometry assays.**

**Supplemental Data Figure S5: Impact of MSC co-culture on macrophage polarization assessed by a CD40, HLA-DR and CD206 staining.**

**Supplemental Data Figure S6: Inhibition of BM-MSC sialylation differentially modulates macrophage polarization towards an M1 phenotype.**

**Supplemental Data Figure S7. Impact of MSC co-culture on macrophage polarization assessed by cytokine productions.**

**Supplemental Data Figure S8. Enhancement of BM-MSC sialylation differentially modulates macrophage polarization towards an M2 phenotype.**
